# Supplementary material for: In-situ spectroscopic probe of the intrinsic structure feature of single-atom center in electrochemical CO/CO2 reduction to methanol
Source: Nat Commun. 2023 Jun 9;14:3401. doi: 10.1038/s41467-023-39153-6 (PMC10256813; doi:10.1038/s41467-023-39153-6)
Supplement: Supplementary file 1 — Supplementary Information [file 41467_2023_39153_MOESM1_ESM.pdf]

Supplementary Information

In-Situ Spectroscopic Probe of the Intrinsic Structure Feature of Single-Atom Center in Electrochemical CO/CO_2 Reduction to Methanol

Xinyi Ren[1,2,‡], Jian Zhao[1,‡], Xuning Li[1,*], Junming Shao[3], Binbin Pan[4,5], Aude Salamé[3], Etienne Boutin[3], Thomas Groizard[3], Shifu Wang[1,6], Jie Ding[7], Xiong Zhang[1], Wen-Yang Huang[8], Wen-Jing Zeng[8], Chengyu Liu[3], Yanguang Li[4,5], Sung-Fu Hung[8,*], Yanqiang Huang[1], Marc Robert[3,9,*] and Bin Liu[7,*]

[1]CAS Key Laboratory of Science and Technology on Applied Catalysis, Dalian Institute of Chemical Physics, Chinese Academy of Sciences, Dalian 116023, China.

[2]University of Chinese Academy of Sciences, Beijing 100049, China.

[3]Université Paris Cité, Laboratoire d'Electrochimie Moléculaire, CNRS, F-75006, Paris, France.

[4]Institute of Functional Nano & Soft Materials (FUNSOM), Soochow University, Suzhou 215123, China.

[5]Jiangsu Key Laboratory for Advanced Negative Carbon Technologies, Soochow University, Suzhou 215123, China.

[6]Department of Chemical Physics, University of Science and Technology of China, Hefei 230026, China.

[7]Department of Materials Science and Engineering, City University of Hong Kong, Hong Kong SAR 999077, China.

[8]Department of Applied Chemistry, National Yang Ming Chiao Tung University, Hsinchu 300, Taiwan.

[9]Institut Universitaire de France (IUF), F-75005, Paris, France.

[‡]These authors contributed equally to this work.

[*]Corresponding Authors:

lixn@dicp.ac.cn (X.L.)

sungfuhung@nycu.edu.tw (S.H.)

robert@u-paris.fr (M.R.)

bliu48@cityu.edu.hk (B.L.)

Supplementary Figures and Tables

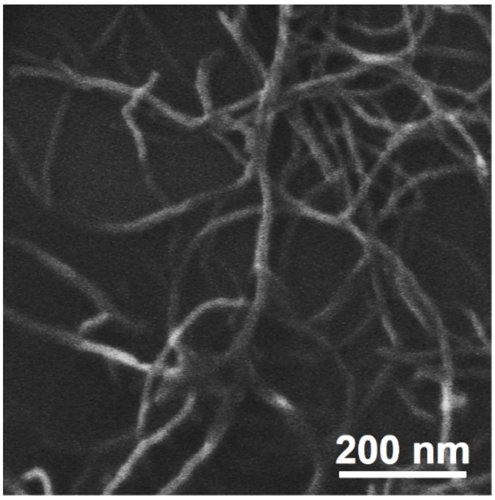

Supplementary Fig. 1. SEM image of pure MWCNTs. The nanotube structure with an average diameter of ~8 nm was observed.

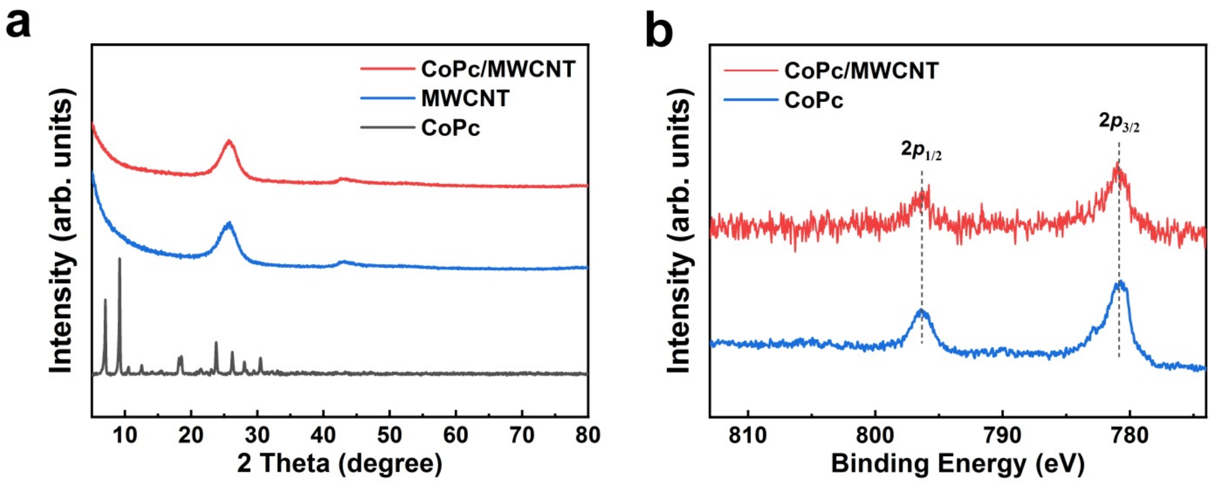

Supplementary Fig. 2. (a) XRD patterns of CoPc/MWCNT, MWCNT and CoPc. (b) Co 2*p* XPS spectra for CoPc/MWCNT and CoPc.

No characteristic peaks associated with CoPc could be observed in the X-ray diffraction (XRD) pattern of CoPc/MWCNT, indicating that the CoPc molecules were uniformly dispersed. In addition, the binding energy of the Co $2p_{3/2}$ peak in the X-ray photoelectron spectroscopy (XPS) spectrum was located at 780.7 eV, corresponding to the +2 valence state of Co in Co–N coordination.

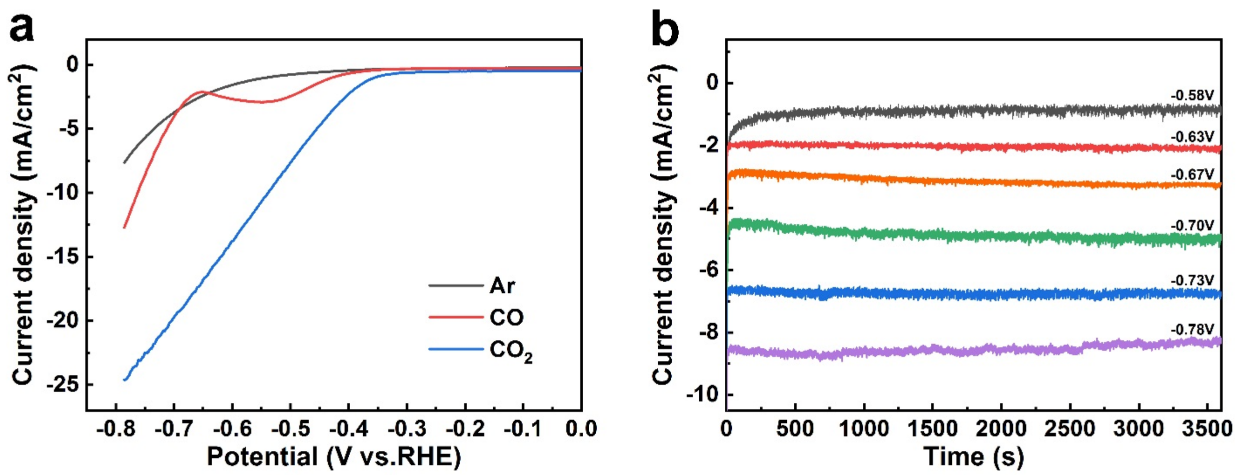

Supplementary Fig. 3. (a) LSV curves of CoPc/MWCNT recorded at a scan rate of 5 mV/s in Ar/CO/CO_2-saturated 0.5 M K_2HPO_4 electrolyte. (b) Time-dependent total current densities at different applied potentials.

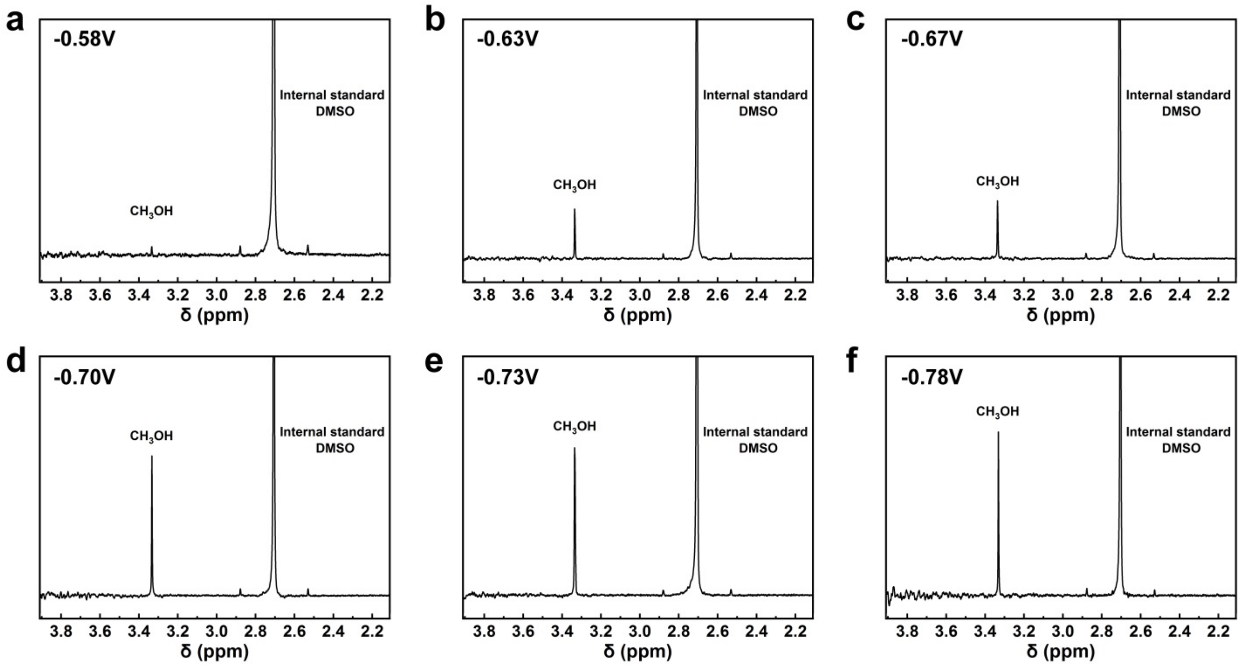

Supplementary Fig. 4. ^{1}H NMR spectra of the electrolyte after CO reduction electrolysis at -0.58 ~ -0.78 V vs. RHE. Methanol was the only detected liquid product and the concentration was calculated by the ratio of CH_3 proton peak area (chemical shift 3.34 ppm) and DMSO internal standard area.

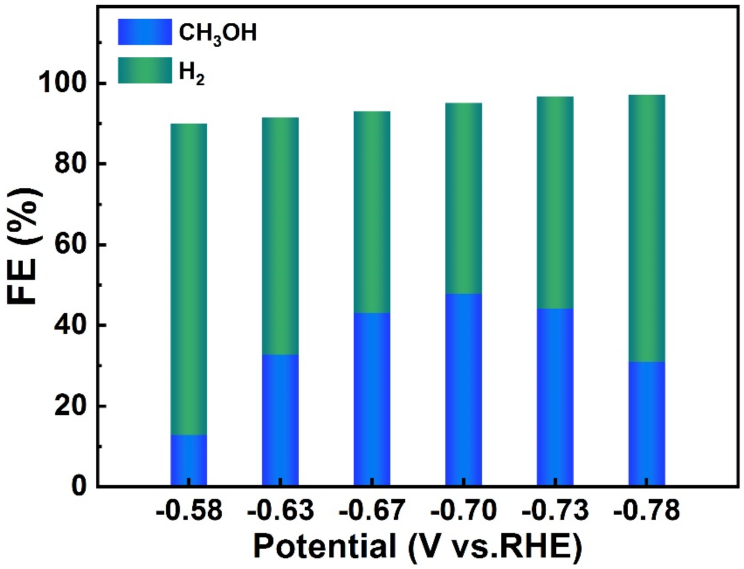

Supplementary Fig. 5. CH_3OH and H_2 Faradaic efficiency under 100% CO at different applied potentials. The total FE of CH_3OH plus H_2 was found to be between 90% and 100% over the entire potential range.

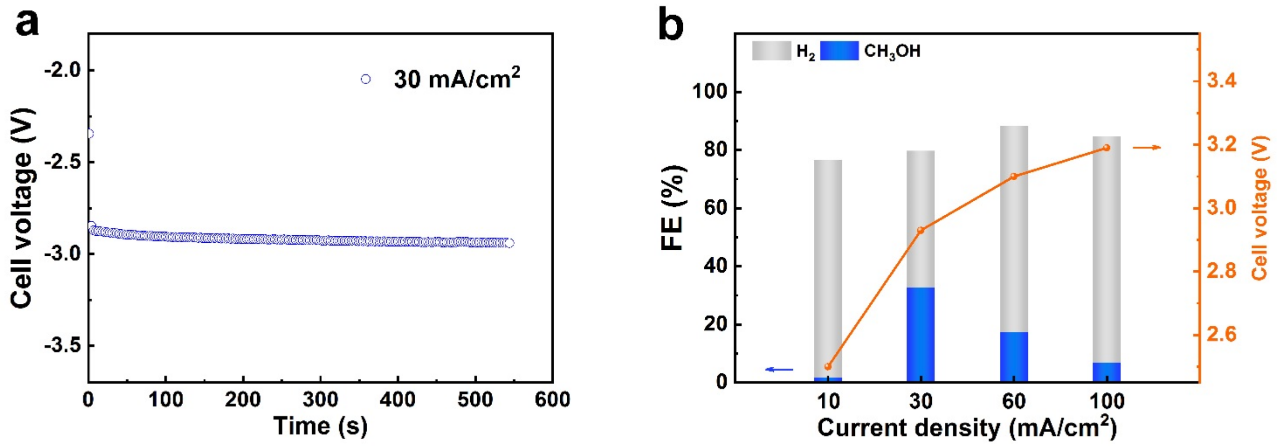

Supplementary Fig. 6. Electrochemical CORR in a membrane electrode assembly (MEA). (a) Chronopotentiometry (*V*-*t*) measurements at the current density of 30 mA/cm^2. Conditions: CoPc/MWCNT (0.3 mg/cm^2) loaded gas diffusion electrode, 10 mL/min flow of 0.25 M K_2HPO_4 aqueous electrolyte, and 40 SCCM flow of CO gas. (b) CH_3OH and H_2 Faradaic efficiency at different current densities in 0.5 M K_2HPO_4. The maximum methanol Faradaic efficiency is 32.7% at the current density of 30 mA/cm^2. Compared with Fig. 2c, the 0.25 M K_2HPO_4 electrolyte is more favorable to improve the methanol Faradaic efficiency, which is most probably due to its better suppression of hydrogen evolution reaction and avoiding the adverse effects of local high alkalinity on methanol production.

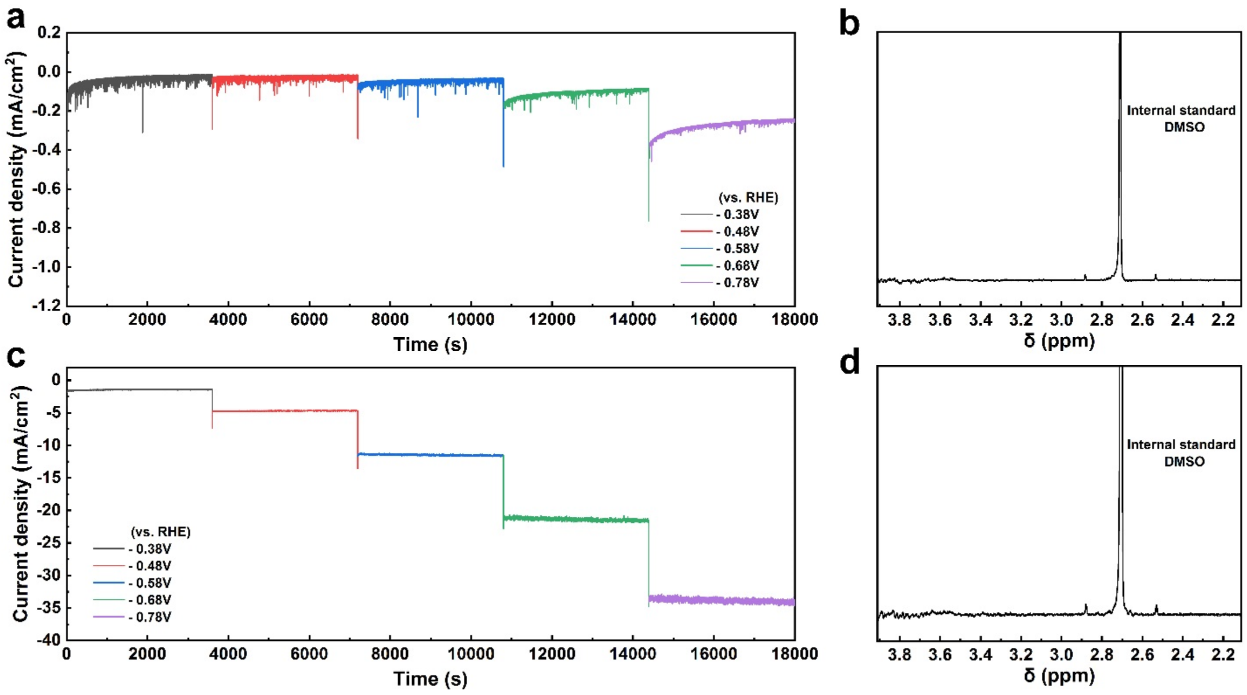

Supplementary Fig. 7. Time-dependent total current densities at different applied potentials of (a) H_2Pc/MWCNT and (c) Co–N_4 single-atom catalyst. ^1H NMR spectra of the liquid products were measured in D_2O and no liquid products were identifiable (b,d). The electrolyte was collected from the chronoamperometry test at -0.38 to -0.78 V vs. RHE for 1 h.

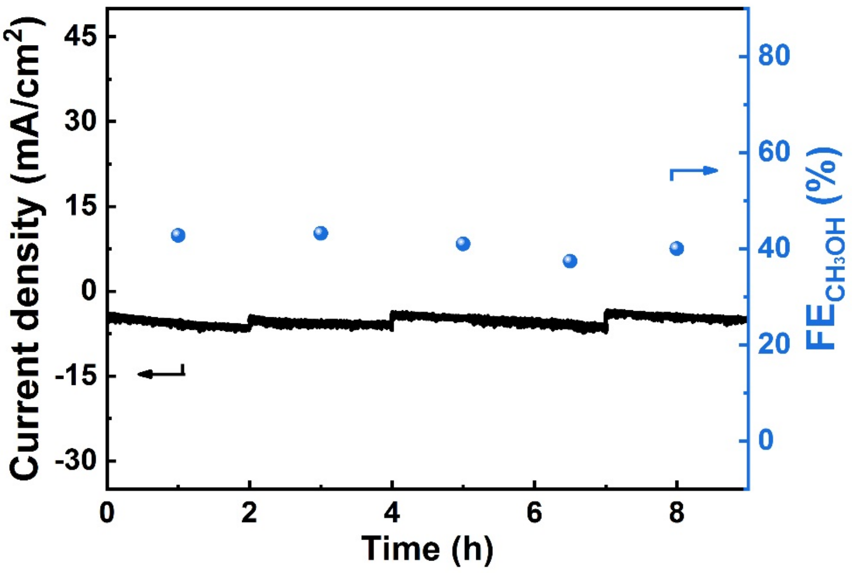

Supplementary Fig. 8. Stability test of CoPc/MWCNT in CO-saturated 0.5 M K_2HPO_4 electrolyte at -0.70 V vs. RHE.

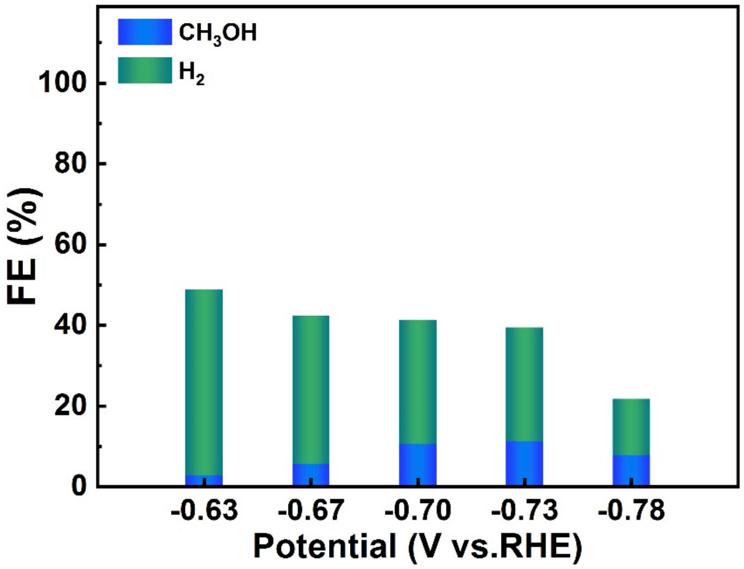

Supplementary Fig. 9. CH_3OH and H_2 Faradaic efficiency under 90% CO + 10% CO_2 at different applied potentials. The total FE of CH_3OH plus H_2 was far lower than ~100% over the entire potential range, suggesting the existence of CO_2-to-CO reaction pathway on CoPc/MWCNT.

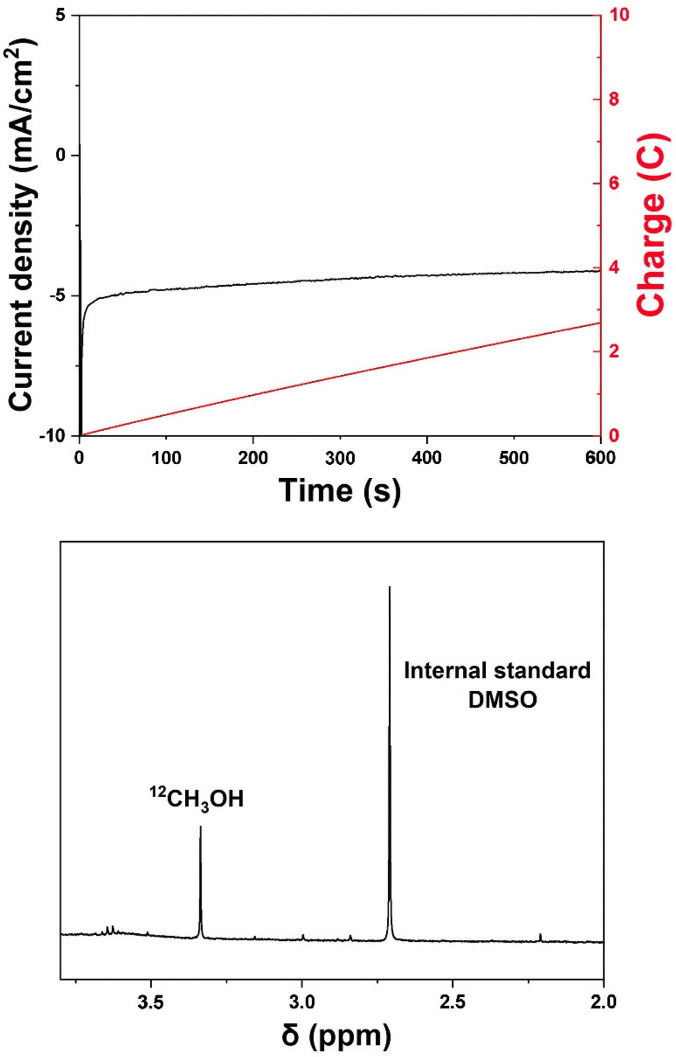

Supplementary Fig. 10. Controlled potential electrolysis of CoPc/MWCNT at *E* = -0.70 V vs. RHE (0.5 M K_2HPO_4) under an almost equimolar amount of $^{13}CO_2$ (1.15 mM) and ^{12}CO (0.95 mM) (gas mixture 96.5% ^{12}CO + 3.5 % $^{13}CO_2$). Top: current trace versus time; bottom: ^1H NMR spectra of the solution taken after 10 min of electrolysis, showing the sole formation of $^{12}CH_3OH$, with a Faradaic yield of ca. 10%.

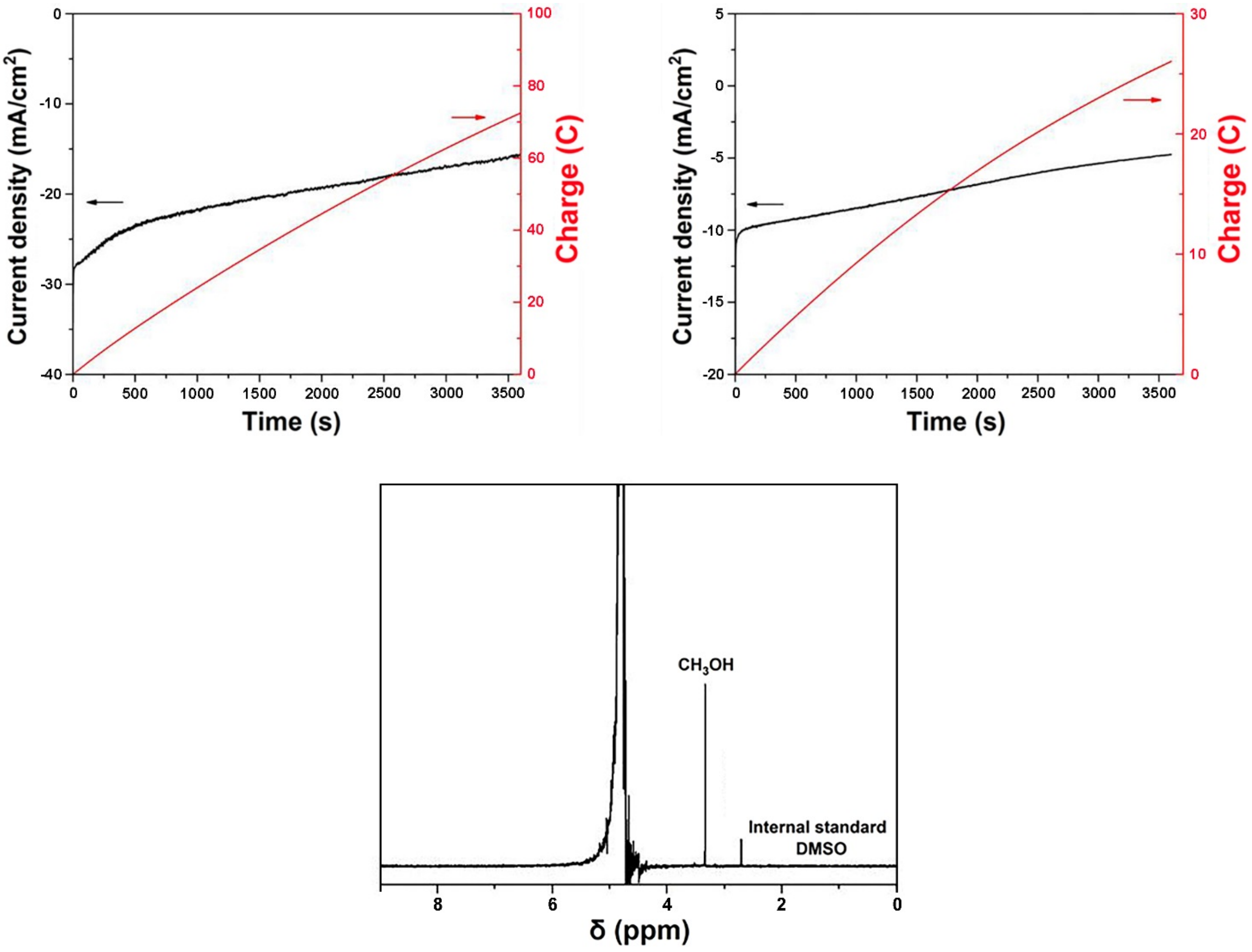

Supplementary Fig. 11. Controlled potential electrolysis of CoPc/MWCNT at *E* = -0.85 V vs. RHE (0.5 M $KHCO_3$, pH 8.5) in a CO_2- (top, left) and CO-saturated (top, right) electrolytes. The electrode preparation is detailed in the Preparation of gas diffusion layer in the Method section. The bottom figure illustrates a typical ^{1}H NMR spectrum obtained after electrolysis (in that case, under CO_2). After 1 h electrolysis, these optimized conditions led to 8% FE_{CH_3OH} (CPE under CO_2 atmosphere) and to 56.7% FE_{CH_3OH} (CPE under CO atmosphere).

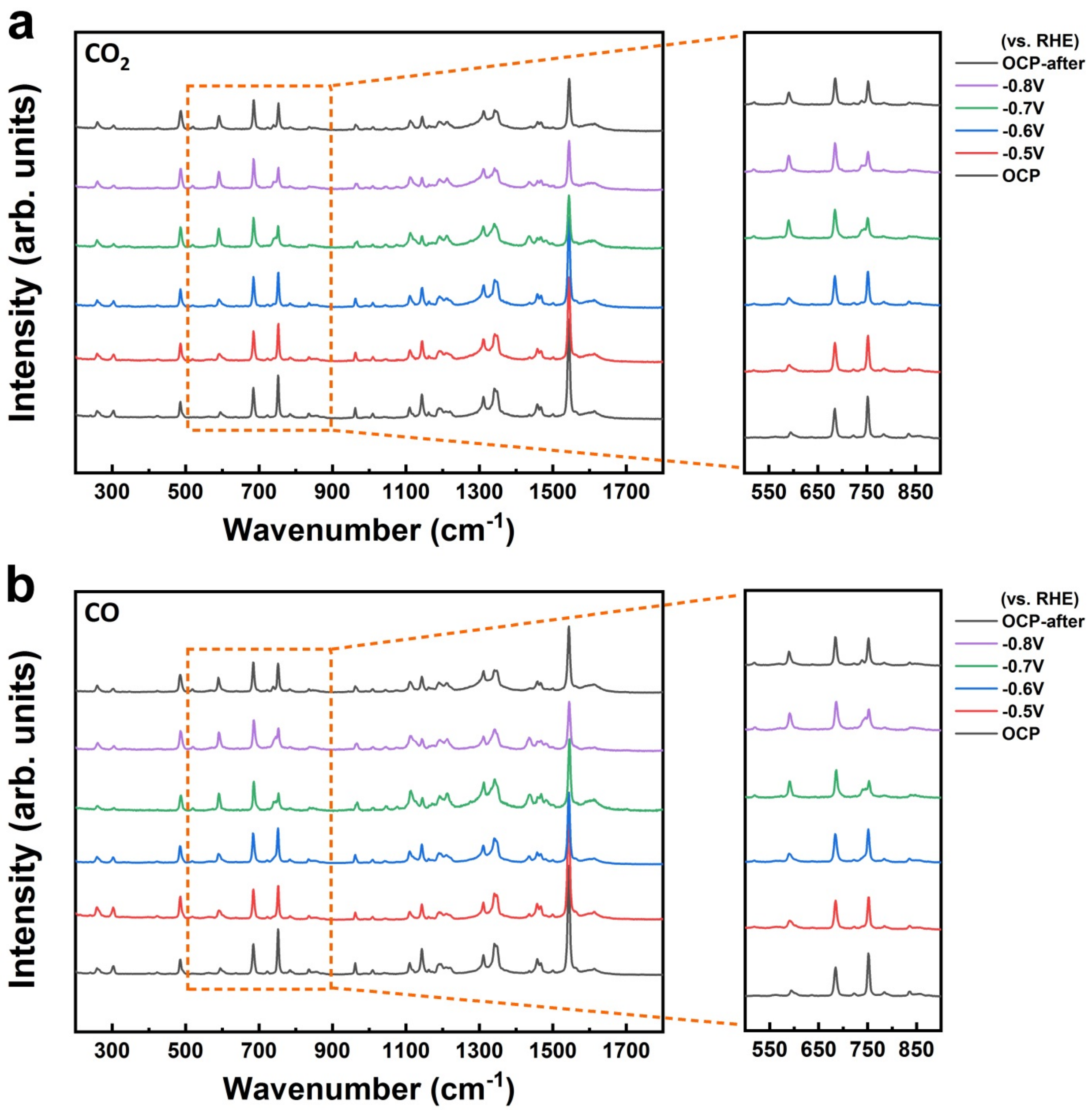

Supplementary Fig. 12. *In-situ* Raman spectra of CoPc/MWCNT measured at OCP and -0.5 V ~ -0.8 V vs. RHE in (a) CO_2 and (b) CO-saturated electrolyte.

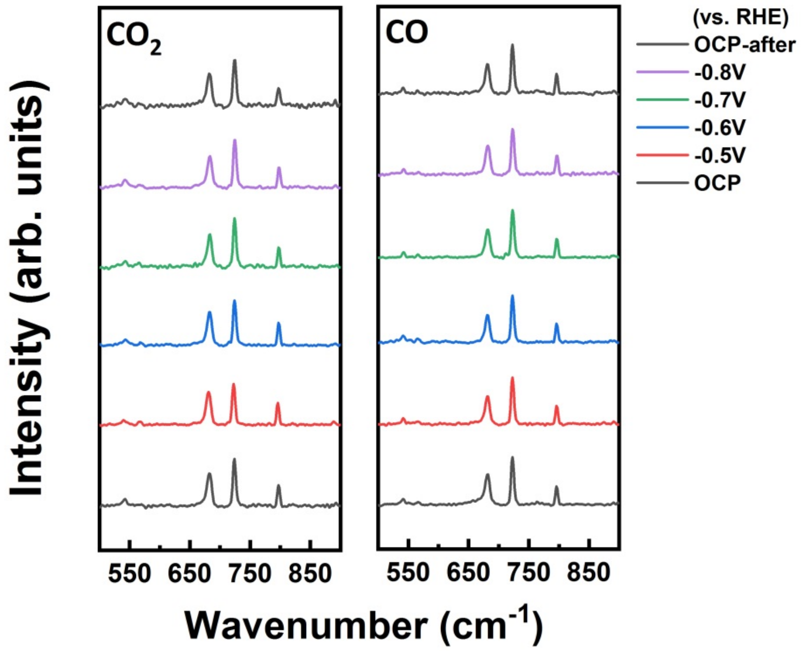

Supplementary Fig. 13. *In-situ* Raman spectra of H_2Pc/MWCNT measured at OCP and -0.5 V ~ -0.8 V vs. RHE in CO_2 and CO-saturated electrolyte.

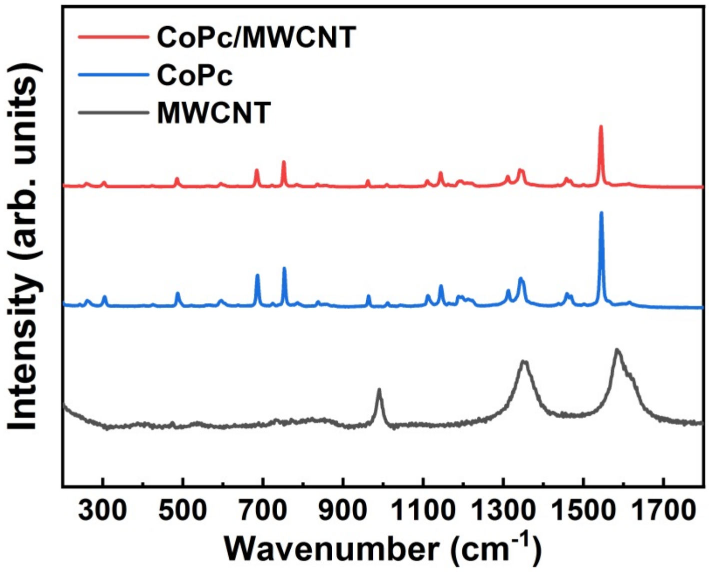

Supplementary Fig. 14. Raman spectra of CoPc/MWCNT, CoPc and MWCNT in 0.5 M K_2HPO_4 electrolyte.

Three characteristic peaks at 1590 cm^{-1}, 1350 cm^{-1}, and 990 cm^{-1} were observed, corresponding to the G peak and D peak of carbon nanotubes, and the adsorption of HPO_4^{2-} ions over carbon nanotubes, respectively. Moreover, signature vibrational peaks in the CoPc/MWCNT spectrum are well consistent with the vibrational features of CoPc molecule, so in the *in-situ* Raman tests, the changes of the peaks on CoPc/MWCNT correspond to the contribution of CoPc.

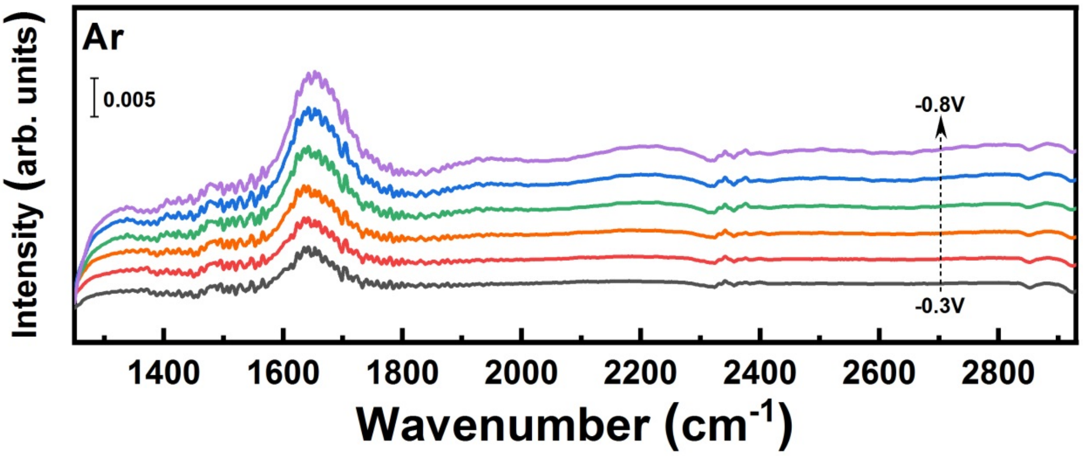

Supplementary Fig. 15. *In-situ* FTIR spectra of CoPc/MWCNT measured at *E* = -0.3 V ~ -0.8 V vs. RHE in Ar-saturated electrolyte. Except for the O–H bending peak of water, no absorption peak of an intermediate was detected.

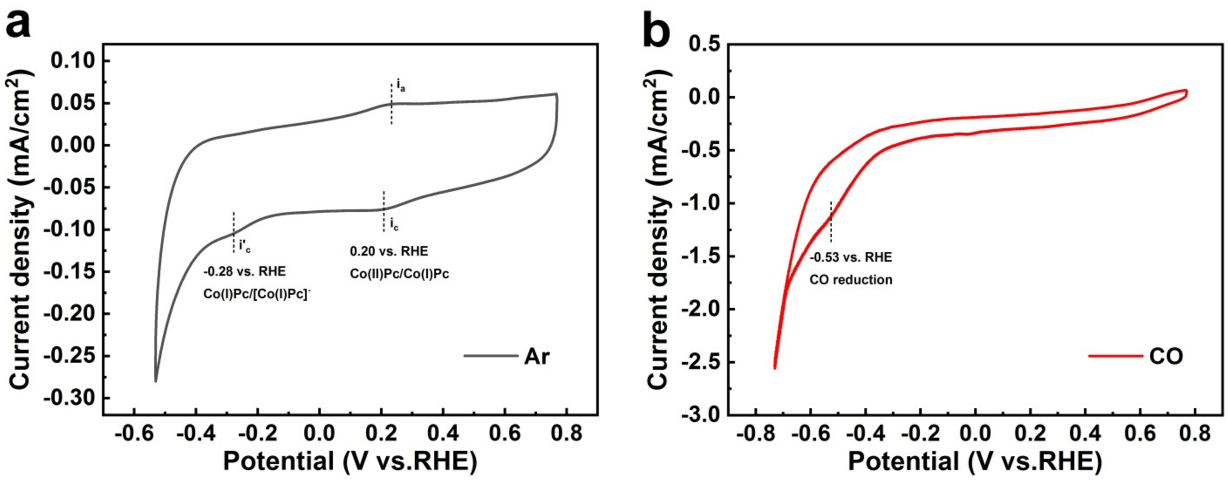

Supplementary Fig. 16. Cyclic voltammetry curves recorded in Ar-saturated (a) and CO-saturated (b) 0.5 M K_2HPO_4 aqueous solution.

As shown, under Ar atmosphere, the first reversible peak at around 0.20 V vs. RHE arises from the reduction of Co(II) to Co(I), while the second reduction peak at around -0.28 V vs. RHE corresponds to the delocalization of the charge obtained onto the macrocycle, suggesting that CO activation took place on the two-electron reduced CoPc center[1,2].

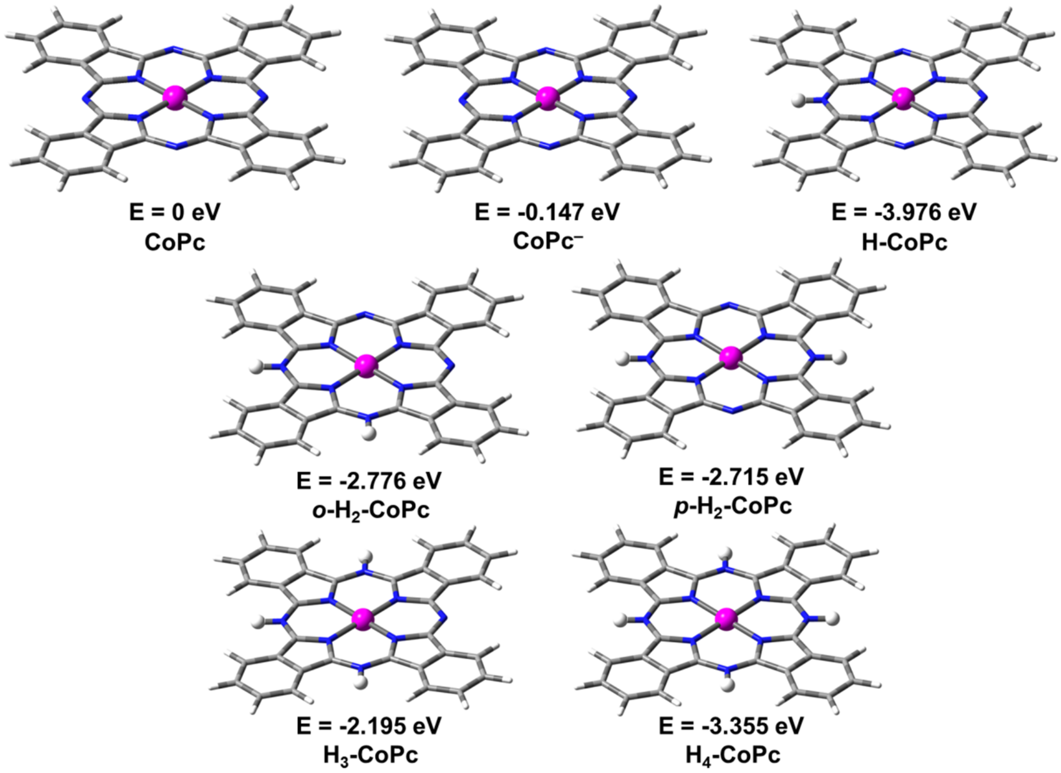

Supplementary Fig. 17. Relative single point energies of CoPc, $CoPc^{-}$, H-CoPc, *o*-H_2-CoPc, *p*-H_2-CoPc, H_3-CoPc, and H_4-CoPc.

The CoPc is regarded as the reference, whose single point energy is set to 0 eV. The energy of H_x-CoPc (x=1-4) relative to CoPc is calculated as $E = E(H_x\text{-CoPc}) - E(\text{CoPc}) - 0.5E(xH_2)$, where the terms on the right side of the equation are the single point energies of H_x-CoPc, CoPc, and H_2, respectively. The calculation results show that H-CoPc exhibits the lowest relative single point energy, indicating that CoPc is easily protonated at the ligand, making it more thermodynamically stable.

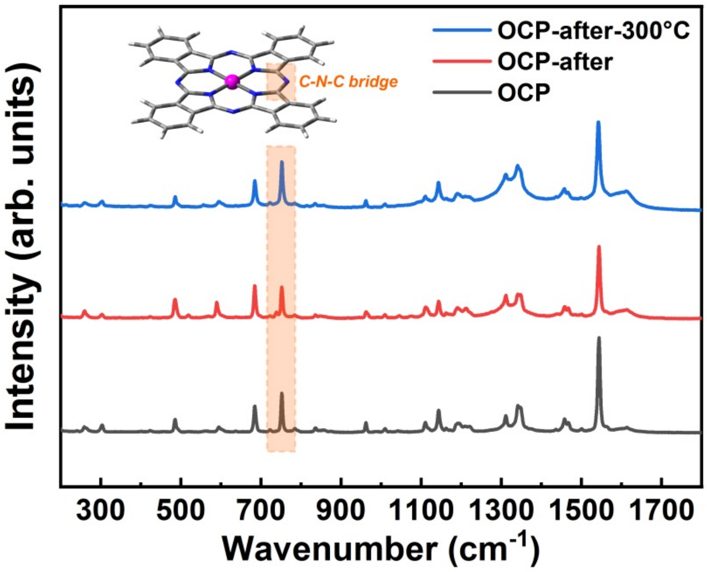

Supplementary Fig. 18. Raman spectra of CoPc/MWCNT at OCP, OCP-after and calcined under Ar atmosphere at 300°C.

The peak at 750 cm^{-1}, which can be assigned to the stretching vibration of M–N bonds and C–N–C bridge bonds in CoPc cannot be fully recovered upon setting back the electrode to OCP, demonstrating that the vibration of the Pc macrocycle is altered during electroreduction. Then, we calcined the carbon paper after reaction at 300 °C under Ar atmosphere, and found that the peak could return to its original state, indicating that the structure of CoPc was slightly changed during electroreduction and hydrogenation of the Pc macrocycle might occur.

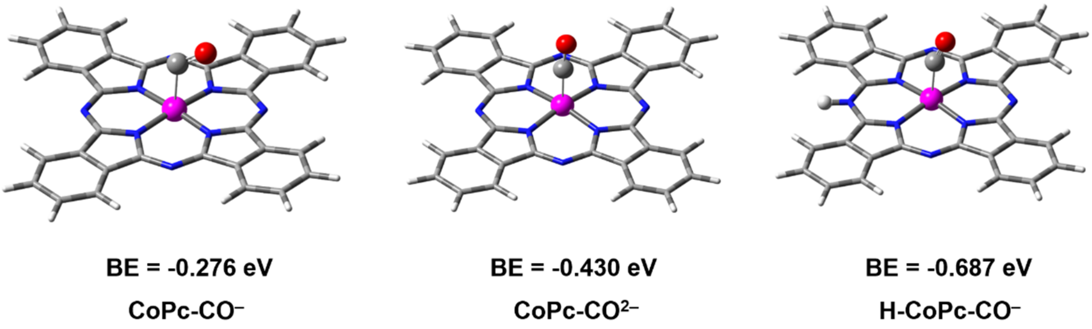

Supplementary Fig. 19. The binding energies of CoPc-CO^-, CoPc-CO^{2-}, and H-CoPc-CO^-.

The results show that H-CoPc-CO^- exhibits the lowest binding energy, indicating that the protonation of Pc can improve the stability of CO binding on CoPc. Hence, H-CoPc functions as the stable structure for CORR/CO_2RR.

H-CoPc	H-CoPc^{-}	H-CoPc-CO_2^{-}
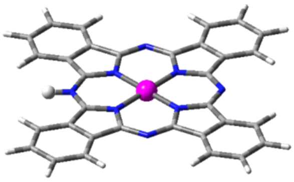	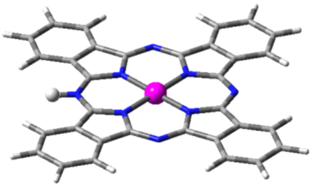	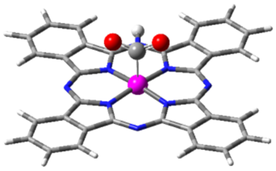<br>H atom on the backside<br>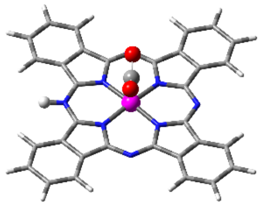<br>H atom in the left direction
**H-CoPc-COOH**	**H-CoPc-CO**	**H-CoPc-CO^{-}**
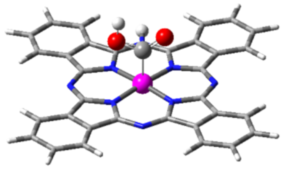<br>H atom on the backside<br>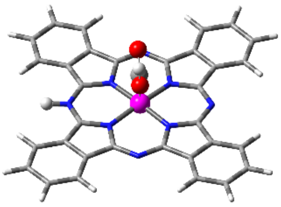<br>H atom in the left direction	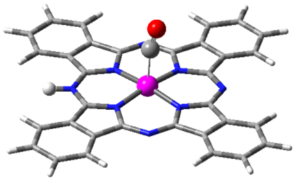	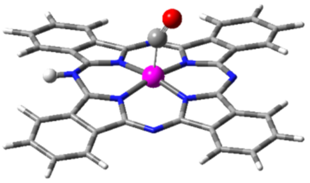
**H-CoPc-CHO**	**H-CoPc-OCH_2**	**H-CoPc-OCH_3**
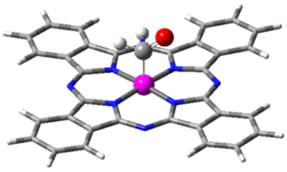<br>H atom on the backside<br>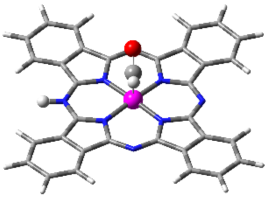<br>H atom in the left direction	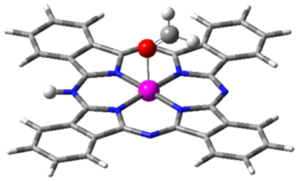	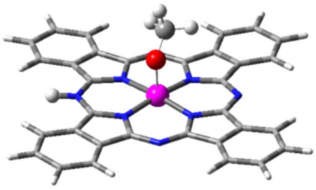

Supplementary Fig. 20. DFT-optimized structure models of H-CoPc and the corresponding

intermediates. The geometries of H-CoPc-CO_2^-, H-CoPc-COOH, and H-CoPc-CHO with the H atom on the backside (top) and in the left direction (bottom) views are provided.

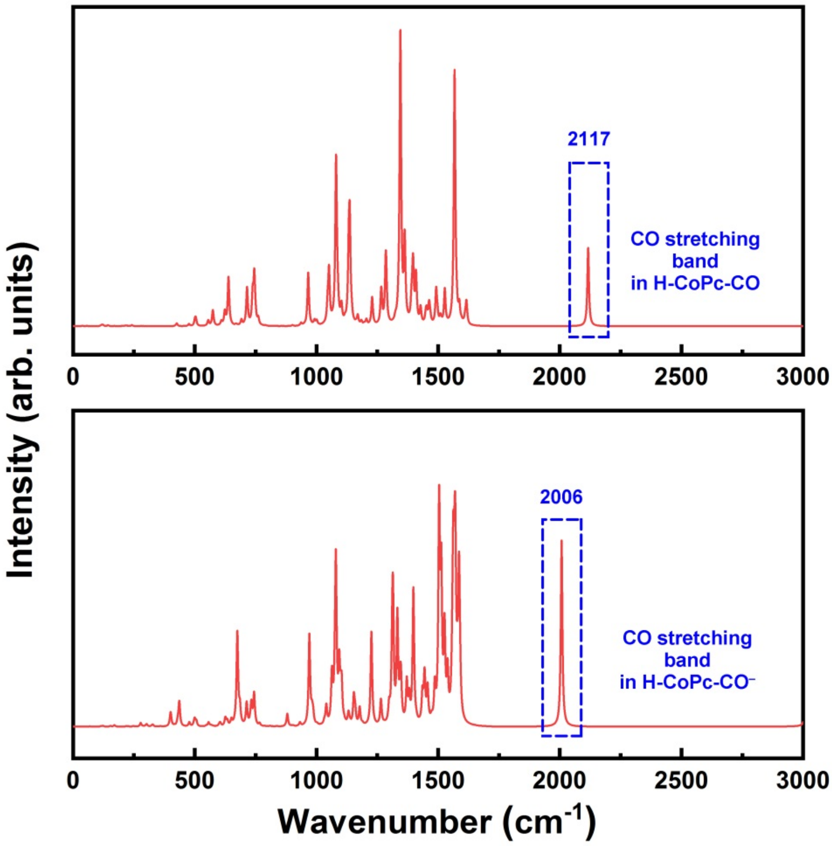

Supplementary Fig. 21. DFT-predicted vibrational frequencies of H-CoPc-CO and H-CoPc-CO^{-} intermediates in FTIR. The peak position of CO stretching in H-CoPc-CO^{-} was located at a lower wavenumber compared to H-CoPc-CO, in agreement with the shift trend observed in *in-situ* FTIR.

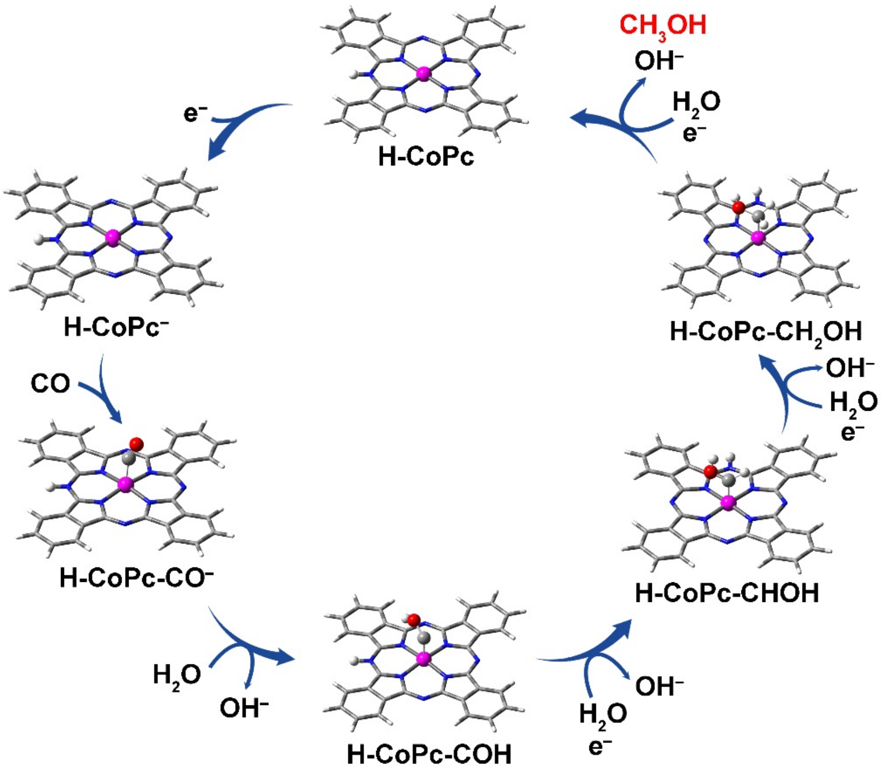

Supplementary Fig. 22. The reaction path for methanol formation via *COH.[3]

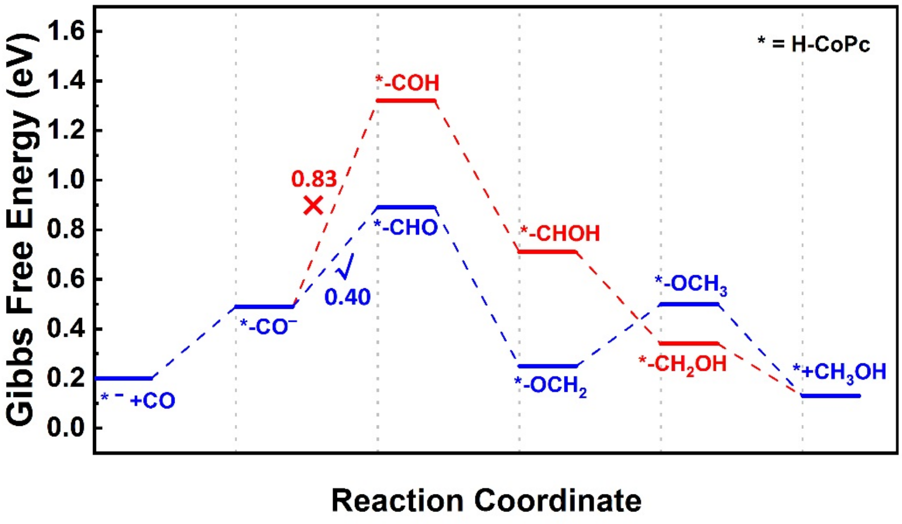

Supplementary Fig. 23. The Gibbs free energies for the reaction path of methanol formation via *COH.

As shown, the Gibbs free energy barrier is 0.83 eV from $*CO^{-}$ to *COH, while 0.40 eV from $*CO^{-}$ to *CHO, elucidating that the reaction path of CO-to-CHO is more preferred.

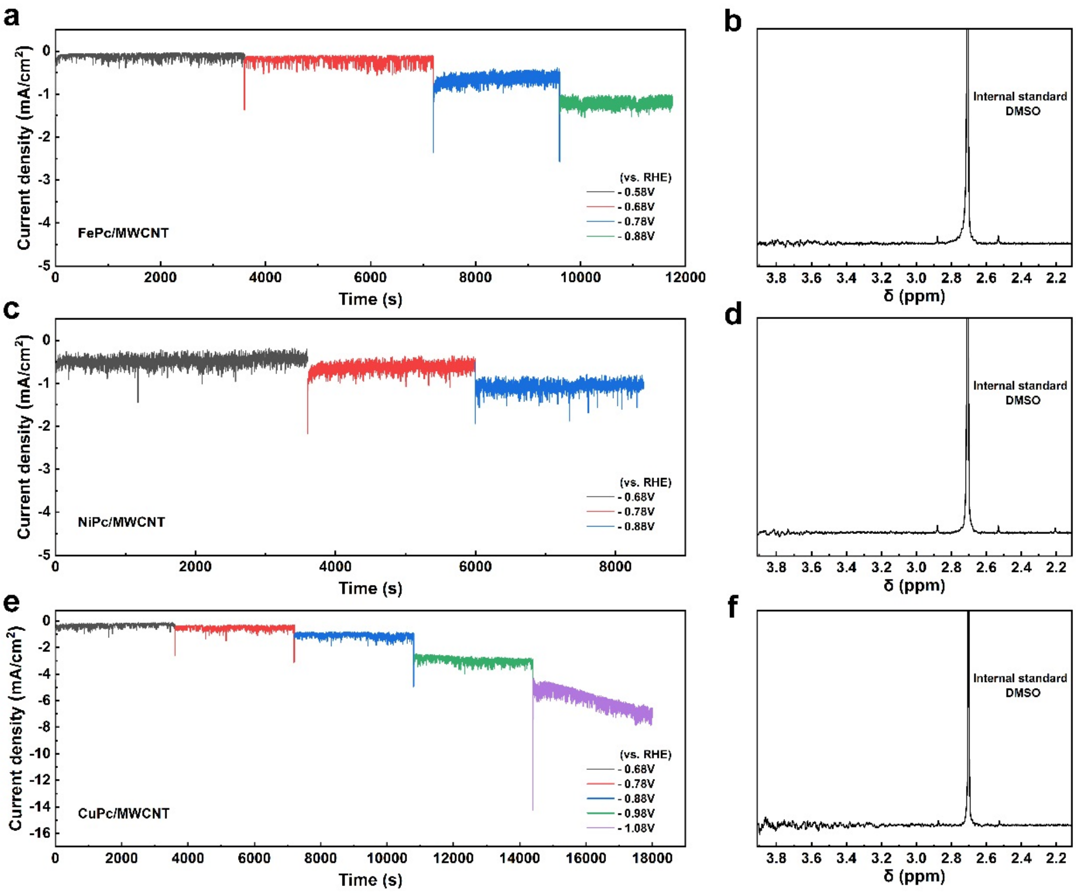

Supplementary Fig. 24. Time-dependent total current densities at different applied potentials for (a) FePc/MWCNT, (c) NiPc/MWCNT and (e) CuPc/MWCNT catalyst. ^{1}H NMR spectra of the liquid products were measured in D_2O and no liquid products were identifiable (b,d,f).

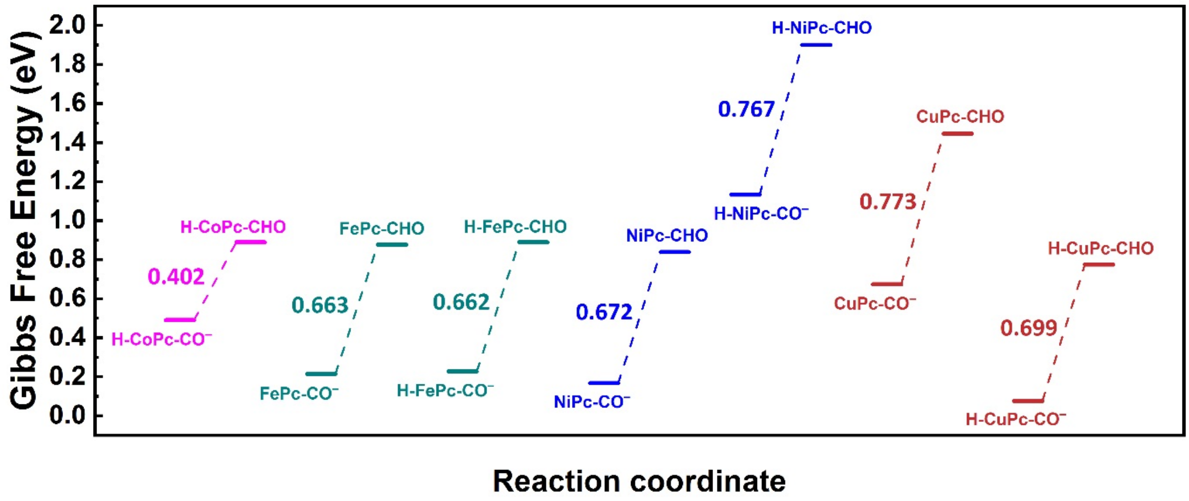

Supplementary Fig. 25. Calculated Gibbs free energy diagrams of $*CO^-$ and *CHO on H-CoPc, FePc, H-FePc, NiPc, H-NiPc, CuPc, and H-CuPc.

We investigated the causes for the inactivity of other metal phthalocyanines using DFT calculations. It can be seen that all other metals have higher energy barriers from $*CO^-$ to *CHO than H-CoPc. Hence, H-CoPc is favored to be used to gain CH_3OH from the theoretical point of view after comparison with other metal phthalocyanine catalysts.

Supplementary Table 1. Bader charge (e) analysis of Co atom in CoPc and the corresponding intermediate models.

Model	CoPc	H-CoPc	H-CoPc^{-}	H-CoPc-CO_2^{-}	H-CoPc-CO	H-CoPc-CO^{-}
q_{Bader}(e)	1.26	0.97	0.89	1.10	1.18	1.03

References

1. Lin, S. *et al.* Covalent organic frameworks comprising cobalt porphyrins for catalytic CO_2 reduction in water. *Science* **349**, 1208–1213 (2015).

2. Leung, K., Nielsen, I. M. B., Sai, N., Medforth, C. & Shelnutt, J. A. Cobalt-porphyrin catalyzed electrochemical reduction of carbon dioxide in water. 2. Mechanism from first principles. *J. Phys. Chem. A* **114**, 10174–10184 (2010).

3. Zhang, J., Cai, W., Hu, F. X., Yang, H. & Liu, B. Recent advances in single atom catalysts for the electrochemical carbon dioxide reduction reaction. *Chem. Sci.* **12**, 6800–6819 (2021).
